# Immunodomination during Peripheral Vaccinia Virus Infection

**DOI:** 10.1371/journal.ppat.1003329

**Published:** 2013-04-25

**Authors:** Leon C. W. Lin, Inge E. A. Flesch, David C. Tscharke

**Affiliations:** Division of Biomedical Science and Biochemistry, Research School of Biology, The Australian National University, Canberra, Australian Capital Territory, Australia; Penn State College of Medicine, United States of America

## Abstract

Immunodominance is a fundamental property of CD8^+^ T cell responses to viruses and vaccines. It had been observed that route of administration alters immunodominance after vaccinia virus (VACV) infection, but only a few epitopes were examined and no mechanism was provided. We re-visited this issue, examining a panel of 15 VACV epitopes and four routes, namely intradermal (i.d.), subcutaneous (s.c.), intraperitoneal (i.p.) and intravenous (i.v.) injection. We found that immunodominance is sharpened following peripheral routes of infection (i.d. and s.c.) compared with those that allow systemic virus dissemination (i.p. and i.v.). This increased immunodominance was demonstrated with native epitopes of VACV and with herpes simplex virus glycoprotein B when expressed from VACV. Responses to some subdominant epitopes were altered by as much as fourfold. Tracking of virus, examination of priming sites, and experiments restricting virus spread showed that priming of CD8^+^ T cells in the spleen was necessary, but not sufficient to broaden responses. Further, we directly demonstrated that immunodomination occurs more readily when priming is mainly in lymph nodes. Finally, we were able to reduce immunodominance after i.d., but not i.p. infection, using a VACV expressing the costimulators CD80 (B7-1) and CD86 (B7-2), which is notable because VACV-based vaccines incorporating these molecules are in clinical trials. Taken together, our data indicate that resources for CD8^+^ T cell priming are limiting in local draining lymph nodes, leading to greater immunodomination. Further, we provide evidence that costimulation can be a limiting factor that contributes to immunodomination. These results shed light on a possible mechanism of immunodomination and highlight the need to consider multiple epitopes across the spectrum of immunogenicities in studies aimed at understanding CD8^+^ T cell immunity to viruses.

## Author Summary

During an infection, the adaptive immune system responds to many epitopes of the pathogen but the strength of these responses varies widely. This unequal distribution of responses across a range of epitopes is known as immunodominance and understanding why it occurs is a fundamental problem in immunology. It is also relevant to vectored vaccines where the intention is to raise immunity against an antigen of choice, but responses to vector epitopes may dominate. We show that the route of infection changes the extent to which the strongest epitope can dominate CD8^+^ T cell responses to vaccinia virus (VACV). The cause of this phenomenon is linked to virus spread and therefore the different lymphoid organs that prime T cell responses for each route. We also show that local draining lymph nodes are sites of more robust competition between T cells compared with the spleen, explaining why immunodominance differs according to route. Finally the normally heightened immunodominance after peripheral VACV infection can be reduced by expression the costimulators CD80 (B7-1) and CD86 (B7-2) from the virus. In summary, we have carefully dissected immunodominance using VACV as a model and in doing so exposed general features of CD8^+^ T cell immunity to pathogens.

## Introduction

Immunodominance is a term used to describe the preferential recognition of some epitopes over others in a complex antigen and is a fundamental property of all immune responses. CD8^+^ T cell responses to viruses are no exception and immunodominance has been noted for many viruses in mice and humans [1], [2]. Immunodominance arises due to factors that affect either 1) the amount of peptide-MHC (pMHC) complexes, including abundance of parent antigen, ease of processing and affinity of peptides for MHC [3]–[17] or 2) the quantity or quality of T cells in the naive repertoire that recognize these pMHC complexes [5], [8], [10], [11], [18]–[29]. An additional determinant that emerges from the intersection of the factors above is immunodomination, which is the ability of T cells with dominant specificities to inhibit responses to less-dominant epitopes. This is observed most clearly in secondary infections, where some memory T cells are clearly less able to compete [30]–[34]. However, it must also operate in primary infection, because deletion of immunodominant epitopes allows responses to subdominant epitopes to increase [10], [30], [35]. Further in some, but not all cases pre-priming of individual epitopes can lead to radically altered dominance hierarchies, presumably because the already primed T cells have an advantage over other specificities [5], [36], [37]. Finally, competition amongst the various clones recognising the same specificity can be directly observed during infection by monitoring the expansion adoptively transferred TCR transgenic T cells compared with the endogenous polyclonal response [38]. While the mechanism of immunodomination remains obscure, it can be relieved if the epitopes are presented on separate antigen presenting cells (APCs). Therefore it is most likely due to competition for resources either on APCs or released by APCs in the immediate environment, but these remain undefined [36], [38]–[40].

Vaccinia virus (VACV) was used as the live vaccine to eradicate smallpox and attenuated strains are now being used as vectors for recombinant vaccines. In understanding both the historical and contemporary usage of VACV, CD8^+^ T cell responses are of interest. Further, VACV provides an excellent model for studies of immunodominance because it has a large genome with many mapped epitopes and infections are entirely acute. These attributes set it apart from the well-studied RNA viruses such as influenza or lymphocytic choriomeningitis viruses, with genomes less than a tenth the size and the herpesviruses, all of which cause latent/persistent infections. Of the roughly 50 CD8^+^ T cell epitopes for VACV identified in the C57BL/6 mouse, B8R_20_ (TSYKFESV) is by far the most dominant [41]–[45]. Depending on the estimate of the total anti-VACV CD8^+^ T cell population, 10–25% of all anti-viral CD8^+^ T cells are specific for this epitope during acute infection and we refer to this as the immunodominant epitope (IDE) [42]. The rest of the mapped epitopes can be considered to be sub-dominant epitopes (SDE) and more than 20 of these induce easily measurable responses that range from 2–0.2% of CD8^+^ T cell at acute times [41]. In the work characterizing the first five CD8^+^ T cell epitopes for VACV in C57BL/6, it was noted that different routes of infection appeared to alter immunodominance with intradermal (i.d.) infection favoring B8_20_, the IDE, over the SDE compared to intraperitoneal (i.p.) infection [42]. Responses to an IDE were also favored by the i.d. route in VACV infections of BALB/c mice [46]. That this phenomenon was observed in two strains of mice (with different sets of epitopes) suggests that it was genuinely linked to immunodominance.

Here we confirm, explore and explain the route-related effects on CD8^+^ T cell immunodominance during VACV infection. We find that immunodominance is linked to the sites of antigen presentation and that individual draining lymph nodes (LN) are environments where there is more competition between T cells of differing specificities than the spleen. Finally we show that the effects of this competition that occurs after i.d. (but not i.p.) infection are reduced by a VACV that expresses the costimulators CD80 and CD86 (B7-1 and 2).

## Results

### Peripheral routes of infection sharpen immunodominance

**Figure 1:**
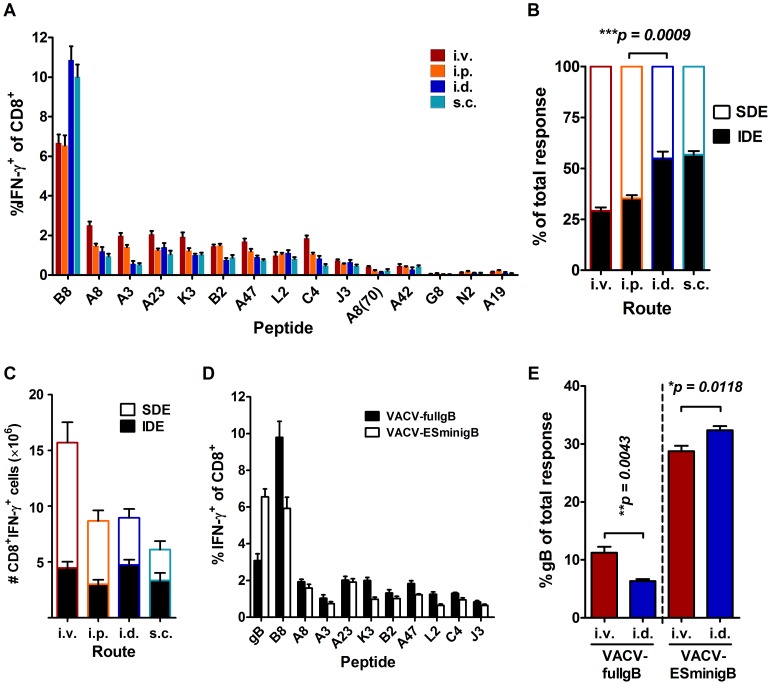
CD8^+^ T cell Immunodominance is altered by the route of infection for VACV. (A–C) CD8^+^ T cell responses to VACV epitopes were determined in C57BL/6 mice infected with 1×10^6^ PFU of VACV WR by i.v., i.p., i.d., or s.c. injection seven days after infection. Data are from two or more experiments and total mice for each route were: i.v. 8, i.p. 16, i.d. 6 and s.c. 10. A) Means and SEM of percent of CD8^+^ T cells responding to each peptide. B) The fraction of all measured responses (sum of responses to all 15 VACV peptides) accounted for by B8_20_-specific (IDE) and the sum of SDE-specific CD8^+^ T cells. C) Responses to VACV epitopes broken down into IDE and SDE as in panel B, but shown as total numbers of CD8^+^IFN-γ^+^ cells per spleen. (D–E) Responses to VACV and HSV gB_498_ epitopes seven days after infection of mice with 1×10^6^ PFU of recombinant VACV-fullgB or VACV-ESminigB. Data are from two experiments with a total of 6 mice per virus. D) Means and SEM of percent of CD8^+^ T cells responding to each peptide after i.v. infection (10 of 14 VACV epitopes shown). E) Response to gB_498_ shown as a fraction of the total measured epitope-specific responses (VACV+gB_498_). Selected statistical significance denoted with p value over appropriate bars.

To extend published results suggesting an effect of vaccination route on CD8+ T cell immunodominance, groups of C57BL/6 mice were infected with 1×10^6^ plaque forming units (pfu) vaccinia virus Western Reserve (WR) strain by i.p., i.d., intravenous (i.v.) and subcutaneous (s.c.) injection. After 7 days, CD8^+^ T cell responses to 15 VACV epitopes (Table 1) were measured using brief *ex vivo* stimulation of splenocytes followed by intracellular staining for IFN-γ (ICS) (Figure 1A). This method allows very accurate enumeration of epitope-specific CD8^+^ T cells at acute times after infection [47]. At a broad scale, the overall hierarchy of the 15 epitopes was similar for the four routes, with B8_20_ being the IDE and the other 14 epitopes being SDEs. However, on closer inspection the previously noted increased dominance of the IDE, B8_20_ over the SDE in i.d. compared with i.p. infected mice was repeated here. There were statistically significant differences for several epitopes (including B8) between i.v., i.p. and i.d. routes, but none differed between i.d. and s.c. infections (statistics not shown). To see the ratio of IDE to SDE responses more clearly, the data were plotted to show B8_20_–specific responses as a fraction of the sum of responses to all epitopes (Figure 1B). When viewed this way, responses in mice infected by the two peripheral routes (i.d. and s.c.) had identical IDE∶SDE ratios of around 50%, but use of the systemic i.p. and to a slightly greater extent i.v. route reduced the dominance of B8_20_ to around 30%. Differences between the peripheral and systemic routes were statistically significant (p<0.01). When the data were analyzed to show the total number of CD8^+^ T cells responding to each epitope, it could be seen that summed responses to the subdominant epitopes are reduced when peripheral routes were used (Figure 1C). The basic phenomenon of skewing towards B8_20_ after i.d. compared with i.p. infection has also been observed in memory CD8^+^ T cell responses, as measured at 28 days after infection and so is not an artifact of the acute infection (not shown).

**Table 1:** Synthetic peptides used in this study.

Name	Sequence	H-2	Gene name
B8_20_	TSYKFESV	K^b^	VACWR190
A8_189_	ITYRFYLI	K^b^	VACWR127
A3_270_	KSYNYMLL	K^b^	VACWR122
A23_297_	IGMFNLTFI	D^b^	VACWR148
K3_6_	YSLPNAGDVI	D^b^	VACWR034
B2_54_	YSQVNKRYI	D^b^	VACWR184
A47_138_	AAFEFINSL	K^b^	VACWR173
L2_53_	VIYIFTVRL	K^b^	VACWR089
C4_125_	LNFRFENV	K^b^	VACWR024
J3_289_	SIFRFLNI	K^b^	VACWR095
A8_70_	IHYLFRCV	K^b^	VACWR127
A42_88_	YAPVSPIVI	D^b^	VACWR167
G8_34_	LMYIFAAL	K^b^	VACWR086
N2_60_	FLMMNKDEL	D^b^	VACWR029
A19_47_	VSLDYINTM	K^b^	VACWR139
gB_498_	SSIEFARL	K^b^	HSV-1 gB
OVA_257_	SIINFEKL	K^b^	ovalbumin

Next we wanted to explore whether this phenomenon was related to the particular selection of epitopes used or would apply to other IDE and SDE when expressed as foreign antigens from VACV. One of the most immunodominant CD8^+^ T cell epitopes mapped to date is the gB_498–505_ epitope (SSIEFARL) of herpes simplex virus glycoprotein B (HSVgB) and we had two recombinant VACVs expressing this epitope. These express full length HSVgB (VACVfullgB) or the gB_498_ epitope as an endoplasmic reticulum-targeted ‘minigene’ (VACV-ESminigB) [48]. Infection of mice with these viruses showed that gB_498_ was a SDE when it required processing from full HSVgB, but became co-dominant with B8_20_ when expressed as an ER-targeted minigene (Figure 1D). These viruses then provided the opportunity to test the effect of route of infection on immunodominance using a single epitope that was either an IDE or a SDE depending on the context. CD8^+^ T cell responses to the gB_498_ epitope were then compared to the sum of responses to all 15 VACV epitopes for the two viruses after i.d. and i.v. infection. In the context of full HSVgB, where gB_498_ ranks as an SDE, responses to this epitope were enhanced roughly two-fold by i.v. compared with i.d. infection, but the opposite result (albeit with a narrower difference) was obtained for the IDE minigene version (Figure 1E). Taken together, the conclusion from these experiments is that VACV infection by peripheral, compared with systemic routes sharpens immunodominance and this can predict which route will maximise responses to foreign antigens expressed from VACV vectors.

### Virus dissemination after infection by different routes

**Figure 2:**
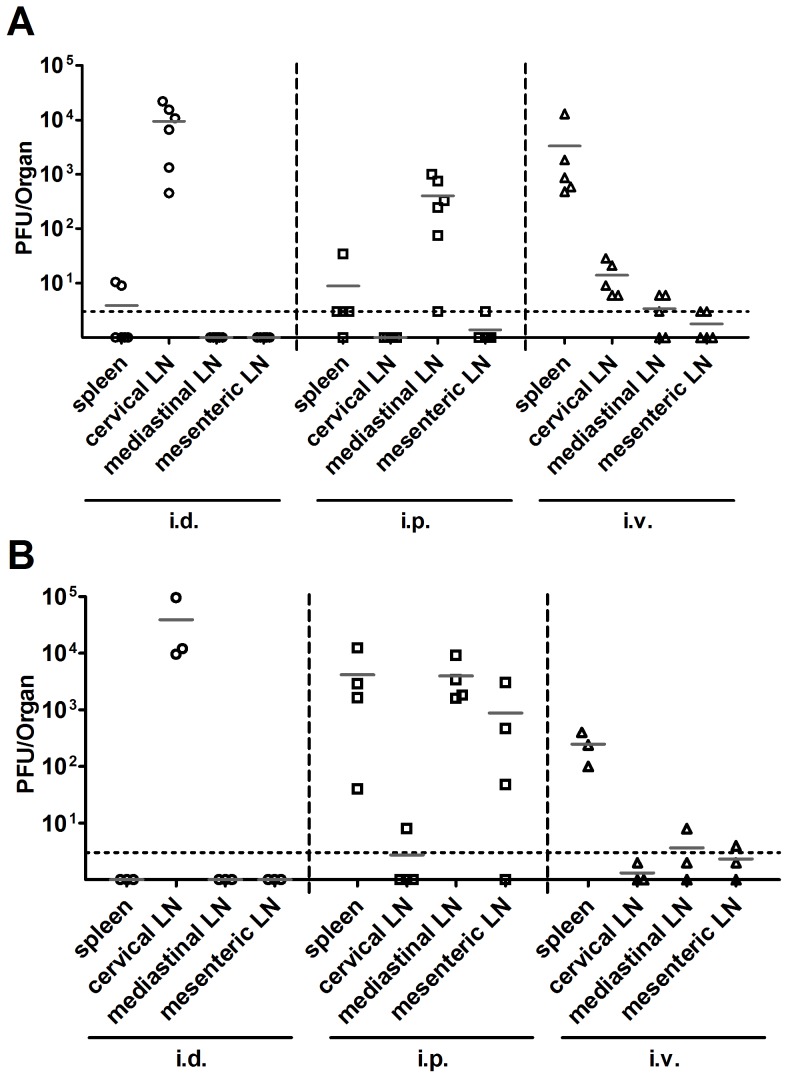
VACV spreads more widely after i.v. or i.p. infection. C57BL/6 mice were infected with 1×10^6^ PFU of VACV WR by i.v., i.p. or i.d. injection. At 6 (A) or 24 (B) hours after infection, spleen and lymph nodes were collected and infectious virus measured by plaque assay. Titers from each organ are plotted with the average for each group shown by a grey bar. The dotted line represents the limit of detection (3 PFU/Organ).

VACV has more opportunity to spread after i.p. and i.v. injection, compared with infection by a peripheral route like i.d. [49]–[51]. We reasoned that virus dissemination might be linked to differences in immunodominance if it affected the range of secondary lymph organs that have access to virus antigen. To test the extent to which the inoculated virus spread to lymph organs, mice were infected by i.d., i.p. and i.v. routes and six hours later, virus titers were measured in cervical, mediastinal and mesenteric LNs and spleen (Figure 2A). As expected, after i.v. injection virus titers were highest in the spleen but virus was also found in each LN. For i.d. and i.p. routes, the highest titers of virus were in the cervical and mediastinal LN respectively, which is consistent with the lymphatic drainage of these injection sites. For i.d. infection, spread of inoculum beyond the cervical LN was seen for two of five mice with low titers found in the spleen. After i.p. infection virus was found in the spleen in four of five and mesenteric LN in one of five mice. Next, spread of virus during the first day of infection was explored by quantifying virus 24 hours after infection by the same three routes (Figure 2B). At this later time point virus spread remained wide after i.v. injection (though titers dropped substantially) and this broad distribution was also seen for mice infected by the i.p. route. In fact the titers were generally higher after i.p. compared with i.v. infection. In contrast, virus was entirely restricted to the cervical lymph node after i.d. infection. These results demonstrate that there was wider spread of virus and potential for antigen presentation after i.v. and i.p. than i.d. infection.

### Systemic routes prime CD8^+^ T cells in more lymphoid organs early after infection

**Figure 3:**
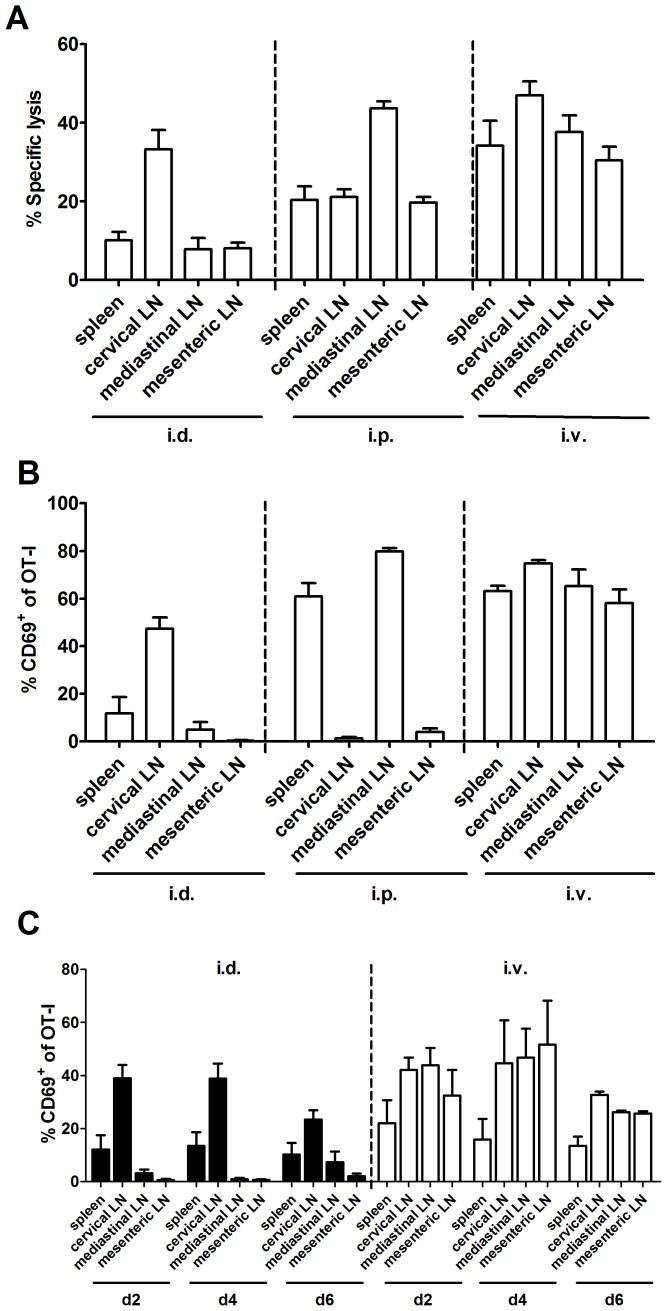
Wider spread but not longevity of antigen presentation is associated with systemic routes. A) Cytotoxicity in lymphoid organs of mice infected with 1×10^6^ PFU of VACV WR by the routes shown was determined by measuring the specific lysis of B8_20_ epitope–pulsed splenocytes two days after infection. Data are from two or more experiments and total mice for each route were: i.d. 7, i.p. 9 and i.v. 7. Killing in the spleen was significantly lower for i.d. compared with i.p. (p<0.05) or i.v. (p<0.01) infected mice. B) B6.SJL mice were given 5×10^6^ OT-I CD8^+^ T cells (CD45.2^+^), then the following day infected with 1×10^6^ PFU of VACV NP-S-GFP by the routes shown. At 24 hours post-infection, expression of CD8, CD45.2 and CD69 were measured by flow cytometry on cells from spleens and LNs. Mean and SEM of percent of OT-I CD8^+^ cells expressing CD69 is shown. Data are from two or more experiments and total mice for each route were: i.d. 5, i.p. 5 and i.v. 3. Priming of OT-I in the spleen was significantly (p<0.05) lower for i.d. compared with i.p. (p<0.01) or i.v. (p<0.001) infected mice. C) B6.SJL mice were infected with 1×10^6^ PFU of VACV NP-S-GFP by the routes shown then given 5 × 10^6^ OT-I CD8^+^ T cells (CD45.2^+^) 1, 3 and 5 days later. Expression of CD8, CD45.2 and CD69 were measured by flow cytometry on cells from spleens and LNs 24 hours after transfer to give times 2, 4 and 6 as shown on the graph. Means and SEM of percent of OT-I CD8^+^ cells expressing CD69 are shown.

Having shown wider spread of virus after i.v. and i.p. routes we wanted to know if this was reflected in the sites where CD8^+^ T cells were primed. The first method used was the *in vivo* cytotoxicity (CTL) assay, which has been used previously to track sites of T cell priming [52]. In the first set of experiments, specific killing of B8_20_-pulsed targets in various lymphoid organs was determined at different times after i.d. infection (Figure S1 in Supporting Information). This showed that at two days after i.d. infection, B8_20_-specific killing activity was already found in the cervical LN, but it took several more days until it was detected at similar levels in other LN and spleen, similar to a report with dermal HSV infection [52]. The killing seen at the other LN and spleen on later days presumably reflects the recirculation of primed CD8^+^ T cells once they leave the site of initial priming. We then examined killing two days after infection by the i.v. and i.p. routes (Figure 3A). Two days after infection by the i.v. route, B8_20_-specific killing was high in all LN and spleen, suggesting priming of CD8^+^ T cells in all these sites. Infection by the i.p. route produced the highest B8_20_-specific killing in the mediastinal LN, but killing was also strong (around 20%) in spleen and other LN. These data suggested a difference in the amount of priming at sites beyond the local draining LN after i.p., compared with i.d. infection and very widespread priming after i.v. injection.

It was unclear in these experiments if the roughly 10% killing in the spleen and non-draining LN after i.d. infection was due to the early migration of some effectors or was background as an artifact of the assay. To look at the sites of priming even earlier, a recombinant VACV (NP-S-GFP) expressing the ovalbumin_257_ (SIINFEKL; OVA_257_) epitope [53], [54] was used in combination with transferred naive OT-I T cells. The dominance of B8_20_ after infection of mice with VACV NP-S-GFP by i.v., i.p. and i.d. routes was similar to that seen for non-recombinant WR (Figure S2 in Supporting Information). To detect the earliest events of priming we looked for CD69 up-regulation on the OT-I cells 24 hours after infection of mice by the three routes (Figure 3B). After i.d. infection, priming of OT-I T cells, as indicated by CD69 up-regulation, was very low in all sites other than the cervical LN. In contrast, i.p. infection primed over 60% of OT-I cells both in the mediastinal LN and the spleen. As expected, i.v. infection was able to prime OT-I in all sites. So by this method, priming of CD8^+^ T cells at sites beyond the local draining LN is efficient after the systemic i.v. and i.p. routes, but not the peripheral i.d. route of infection. While we saw differences in the spread of priming sites, it remained possible that the length of antigen presentation also differed and that this might drive changes in immunodominance. To test this we used the OT-I model again, but this time did transfers at various times after infection (day 1, 3 and 5) by i.d. or i.v. route and looked for CD69 up regulation 24 hours later (Figure 3C). Overall, there was no difference in kinetics of presentation between the routes and priming of OT-I was observed on all days, though it began to wane at the latest time (day 5 to 6). However, we were surprised to see that priming in the spleen was relatively poor at all times after i.v. infection in this experiment, which was in contrast to findings earlier after infection (Figure 3B). To ensure that this was sound, an experiment was done to include a day 1 readout of OT-I activation side-by-side with later times after i.v. infection and this confirmed that priming in the spleen declines more rapidly than in LN (not shown). Interestingly, by day 3–4, there is little difference in priming in the spleen between the i.v. and i.d. infected mice. All together these experiments show that a difference between the systemic and peripheral routes is the extent to which antigen spread allows CD8^+^ T cell priming to take place at early times in lymphoid organs beyond the LN and perhaps especially in the spleen.

### Reduced immunodominance is associated with robust priming in the spleen

**Figure 4:**
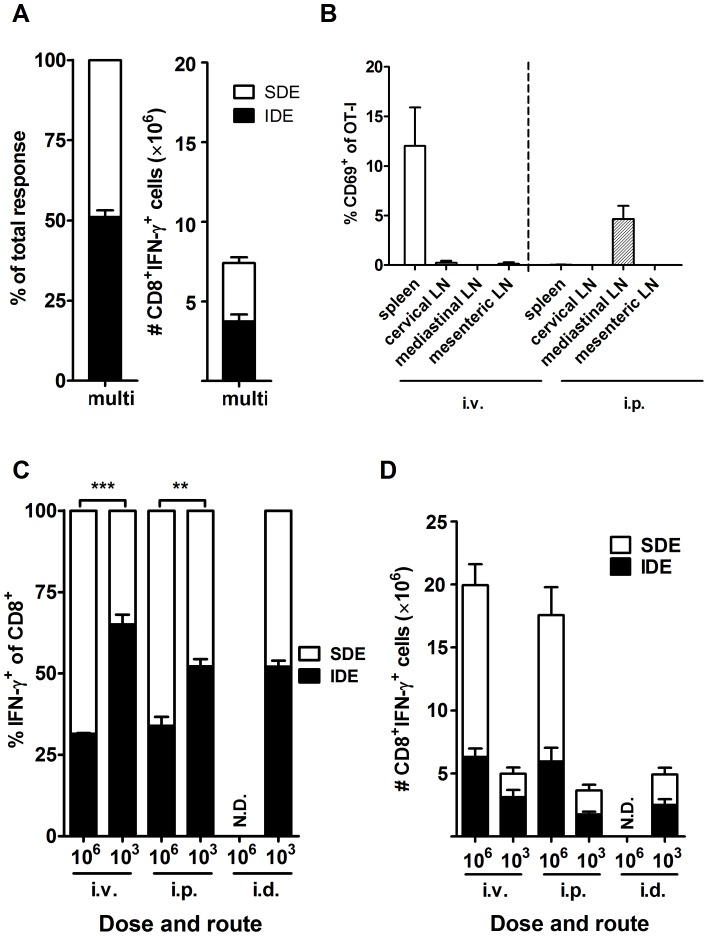
Immunodominance is reduced by robust priming in the spleen. A) CD8^+^ T cell responses to 15 VACV epitopes were measured by intracellular staining of IFN-γ seven days after infection with 1×10^6^ PFU of VACV WR in total spread equally across four peripheral sites (left and right ear pinnae, i.d. and shanks, s.c.). The fraction of the total measured response accounted for by the IDE-specific and the sum of SDE-specific CD8^+^ T cells is shown on the left. On the right, responses are again broken down into IDE and SDE, but shown as total numbers of CD8^+^IFN-γ^+^ cells per spleen. Mean and SEMs of 5 mice from two experiments are shown. B) Priming of OT-I CD8^+^ T cells as demonstrated by upregulation of CD69 expression in the lymphoid organs shown 24 hours after infection with 1×10^3^ PFU (low dose) of VACV NP-S-GFP by i.v. or i.p. injection (as for figure 3B). Data are from more than two experiments; 4 mice for i.p. and 3 for i.v. route. C-D) CD8^+^ T cell responses to 15 VACV epitopes in mice infected with 1×10^3^ or 1×10^6^ PFU of VACV WR by the routes shown, as described previously. Data are from more than one experiment and numbers of mice were: i.v. 10^6^ = 3, 10^3^ = 7; i.p. 10^6^ = 5, 10^3^ = 18; i.d. 10^6^ = not done (N.D.), 10^3^ = 11. C) Data shown are the fraction of the total measured response accounted for by the IDE-specific and the sum of SDE-specific CD8^+^ T cells. D) Responses are again broken down into IDE and SDE, but shown as total numbers of CD8^+^IFN-γ^+^ cells per spleen. Statistical significance denoted with p value over appropriate bars.

Next we wanted to explore whether the number of priming sites and/or levels of presentation lead to reduced immunodominance. First, peripheral routes were explored and priming in more LN was achieved by simultaneous injections (two i.d. and two s.c.) at four different sites. Seven days later, responses to the panel of 15 VACV peptides were determined. In mice that received these multiple concurrent injections, responses to the VACV epitopes were similar to those in mice infected at a single site, with B8_20_–specific CD8^+^ T cells accounting for 50% of the total epitope-specific response (Figure 4A and compare with Figure 1B and C). This suggested that if priming was restricted to LN, increasing the number of lymphoid organs where priming occurs does not reduce immunodominance. In the second experiment, the dose of virus given was reduced to 1×10^3^ PFU, which we reasoned would stop the spread of virus to the spleen after i.p. injection and also greatly limit the number of APCs irrespective of route. First, VACV NP-S-GFP and OT-I transfer was used to directly examine sites of priming after this low-dose infection (Figure 4B). At 24 hours after i.p. infection, only modest priming (5% of OT-I activated) was observed with this dose and it was exclusively in the mediastinal LN. When a similar low dose was used by the i.v. route, again priming was poor (10% of OT-I), but in this case it was found only in the spleen. Therefore the reduced dose clearly limited the spread of antigen and the levels of presentation. Next we examined the effect of this reduced dose on immunodominance and found that mice infected with a low-dose by the i.p. or i.v. routes had CD8^+^ T cell responses more heavily dominated by the IDE, which now accounted for 50% and 60% of the epitope-specific response respectively (Figure 4C). This is similar, if not more extreme domination by B8_20_ as seen for i.d. infection at the standard high dose. By contrast, reducing the dose to 1×10^3^ PFU in i.d. infection did not further sharpen immunodominance for this route. The lower dose given by all routes resulted in much lower total numbers of epitope-specific CD8^+^ T cells and again in the case of i.p. and i.v. routes it was SDE-specific responses that were most reduced (Figure 4D). For example reducing dose in the i.v. route roughly halved the number of B8_20_-specific CD8^+^ T cells, but reduced the number of SDE-specific T cells by around three quarters. In case the dose changed the kinetics of response for the various epitopes, the immunodominance hierarchy was examined at days 6, 7, 8, 9 and 11 after i.p. infection and the dominance of B8_20_ was remarkably stable across these five times (Figure S3 in Supporting Information). From these data it was concluded that simply increasing the number of lymph organs where priming occurs does not reduce immunodominance, neither does priming in the spleen guarantee this outcome. Rather, only where priming in the spleen is substantial can immunodominance be reduced. This suggests a requirement for a higher number of APCs being involved in priming in one organ (as offered by the spleen), rather than there being a special property of splenic versus LN APCs.

### Immunodomination is greater after peripheral infection

**Figure 5:**
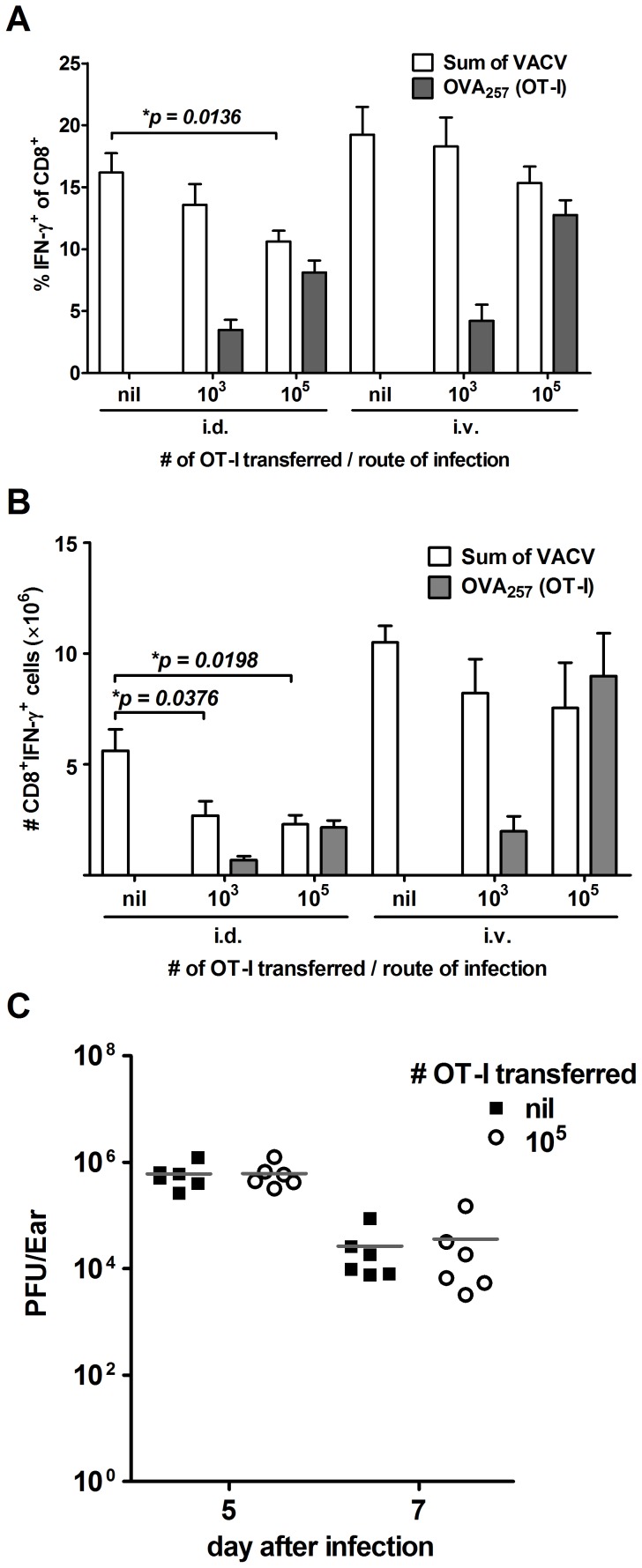
Greater cross-competition from transferred OT-I cells after peripheral infection. A–B) B6.SJL mice were infected with 1×10^6^ PFU of VACV NP-S-GFP by i.d. or i.v. injection a day after receiving the numbers of OT-I CD8^+^ T cells as shown. Seven days later, CD8^+^ T cell responses to 15 VACV epitopes plus OVA_257_ were measured by intracellular staining of IFN-γ. OT-I T cells were identified by expression of CD45.2. CD8^+^ T cells responding to VACV (as a sum of all 15 peptides) and OT-I CD8^+^ T cells responding to OVA_257_ are show as a percent of all CD8^+^ T cells (A) and as a total number of CD8^+^ IFN-γ^+^ cells per spleen (B). Graph shows the average ± SEM and data come from two independent experiments and a total of at least 4 mice for each route and dose of OT-I cells. Statistical significance denoted with p value over appropriate bars. C) B6.SJL mice that received the number of OT-I CD8^+^ cells as shown were infected with 1×10^6^ PFU of VACV NP-S-GFP i.d.. On day 5 and 7 post-infection, virus titers in the ear pinnae were determined by plaque assay. Data are titers from individual ears and the grey bar represents the average.

The results thus far suggested that the sharpened immunodominance after i.d. infection could be due to a limiting resource needed for priming (e.g. number of APCs) in LN and therefore greater potential for immunodomination. To examine immunodomination directly, cross competition between transferred OT-I cells responding to OVA_257_ expressed from VACV NP-S-GFP and endogenous CD8^+^ T cells responding to the VACV epitopes was examined. This approach was originally used to examine competition between T cell clones for OVA_257_, but OT-Is can also cross-compete with native virus epitopes co-expressed with OVA_257_ [38], [55]. Two doses (1×10^3^ and 1×10^5^) of congenically marked OT-I T cells (CD45.2^+^) were transferred into B6.SJL mice (CD45.1^+^), which were infected with VACV NP-S-GFP a day later. After seven days, splenic responses to the set of 15 VACV peptides (CD45.1^+^) and the number of responding OT-I T cells (CD45.2^+^) were measured by ICS. This experiment was done with mice infected by the i.d. route and the i.v. route to represent the greatest and least domination by B8_20_ in the experiments shown thus far. When data were analyzed either as a percent of CD8^+^ T cells (Figure 5A) or as total number of CD8^+^ T cells (Figure 5B), responses to VACV epitopes were significantly reduced by the presence of competing OT-I T cells after i.d., but not i.v. infection. This is despite the relatively larger expansion of the transferred OT-I cells in i.v. infected mice. No significant suppression of VACV-specific responses by OT-I transfer was seen when mice were infected with a control virus that did not express the OVA_257_ peptide (Figure S4 in Supporting Information). It was possible that the transfer of so many OT-I cells might influence the amount of virus growth and therefore antigen presentation during i.d. infection. This was tested by removing the ears from i.d. infected mice that had received OT-I or no transferred cells and determining virus titers by plaque assay. There was no difference in the amount of infectious virus found in ears of mice that received OT-I cells, compared with controls (Figure 5C). Together these data suggest that CD8^+^ T cell responses are more prone to immunodomination after i.d., compared with i.v. infection.

### Costimulation reduces immunodominance after i.d. but not i.p. infection

**Figure 6:**
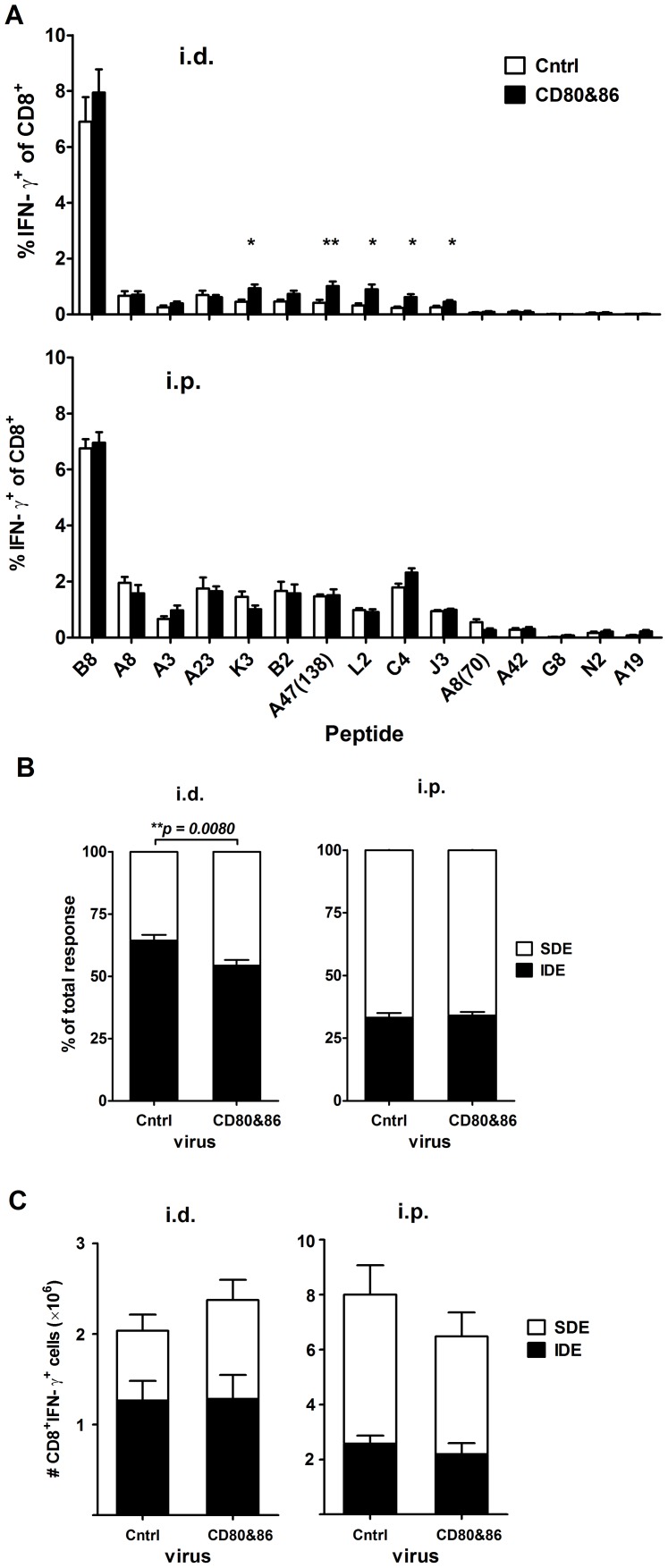
Immunodominance in i.d. but not i.p. infected mice was reduced by over-expression of CD80 and CD86. CD8^+^ T cell responses to VACV epitopes were determined in C57BL/6 mice infected i.d. with 1×10^4^ PFU of VACV-CD80&86 or control virus (VSC-8) or i.p. with 1×10^6^ PFU of the same viruses. Seven days later, CD8^+^ T cell responses to 15 VACV epitopes were measured by intracellular staining of IFN-γ. Data are from two experiments and a total of 8 and 6 mice per virus for i.d. and i.p. respectively; route is shown above each graph. A) Means and SEM of percent of CD8^+^ T cells responding to each peptide. * p<0.05, ** p<0.01. B) The fraction of all measured responses accounted for by B8_20_-specific (IDE) and the sum of SDE-specific CD8^+^ T cells. Statistical significance denoted with p value over appropriate bars. C) Responses to VACV epitopes broken down into IDE and SDE as in panel B, but shown as total numbers of CD8^+^IFN-γ^+^ cells per spleen.

Immunodomination is most likely the result of T cells competing for limited resources on or very close to APCs, but none have been identified. One essential resource required for CD8^+^ T cell priming that has been proposed, but not shown to be involved in immunodominance is costimulation [56]–[58]. These studies used mice deficient in CD28 or treated mice with soluble reagents that block the interaction between CD28 and the costimulators CD80 (B7-1) and CD86 (B7-2). However, this approach only tests the effect of eliminating costimulation and so cannot reveal whether costimulation under normal conditions can be limiting and might be a resource for which CD8^+^ T cells compete. To test this possibility, we used a recombinant VACV expressing CD80 and CD86 (VACV-CD80&86) and infected mice by the i.d. and i.p. routes. Priming of CD8^+^ T cells by VACV is thought to be largely via direct presentation, so this virus should increase the amount of costimulation available on each APC [53], [59], [60]. Consistent with a role for limiting CD80 and CD86 playing a role in immunodomination, mice infected i.d. with VACV-CD80&86 had higher responses to SDEs and the ratio of IDE to SDE was significantly lower than in mice infected with the control VACV (Figure 6). By contrast, in the i.p. infected mice there was no enhanced response to any epitope and immunodominance was unaltered. Further when analyzed by total number of VACV-specific CD8^+^ T cells, in mice infected by the id. but not i.p. route expression of CD80 and CD86 improved the sum of responses to SDEs, but not to B8_20_. From these results we conclude that expression of costimulators by a recombinant VACV reduces immunodomination.

## Discussion

Infection route has been suggested to alter several aspects of CD8^+^ T cell responses to a variety of viruses including priming mechanism, magnitude and quality [61]–[65]. Here we demonstrate clearly that for VACV, immunodominance also needs to be considered. This leads to the first important conclusion of this work, which is that where magnitude of response is a primary read-out, examining responses to a single epitope can be misleading. For example, if B8_20_ was used as a sole epitope in the experiment shown in Figure 1A, one would conclude that i.d. or s.c. injections of VACV were most immunogenic. In contrast if the majority of the SDE were chosen, the opposite conclusion would be drawn. The size of differences in response for individual SDE varied, but were up to four-fold for C4_125_ across the routes. Analyzing the total number of epitope-specific CD8^+^ T cells removes the apparent improvement of B8_20_-specific response seen by the peripheral routes, but makes the suppressive effect of these routes on the SDE more pronounced, with the difference for C4_125_ being more than seven-fold. So while as an overall picture, the change in dominance profile across the doses seems quite modest compared with the very big differences in virus spread and number of priming sites, effects were substantial for some individual epitopes. Much VACV immunology in the past has used recombinant viruses and responses to a single foreign epitope were monitored. In the light of our results some conclusions from these earlier experiments may need to be reconsidered. Indeed the experiments shown here with HSVgB_498_ demonstrate that different forms of antigen can change the dominance ranking of an epitope dramatically in the context of a recombinant VACV. This in turn alters its competitiveness as an immunogen differentially according to route. There are also lessons here for studies of immunodominance using other viruses where few epitopes are known or used.

The original experiments that indicated a role for route in VACV immunodominance used only dermal scarification and i.p. routes [42]. By including i.v. and s.c. routes here, the association between virus spread and immunodominance was noted and then confirmed virologically. The lack of spread after i.d. infection was not surprising [49]. However, the much larger amount of virus found in all organs 24 hours after i.p., compared with i.v. injection was less expected. The reduction in virus in all lymph organs from 6 hrs to 24 hrs after i.v. injection suggests that these sites do not sustain VACV replication. Therefore the higher amounts of virus found a day after i.p. injection are most likely the result of continued draining of virus generated at other sites, rather than infection of the lymph organs. This is an advantageous feature of the VACV model in that findings made in lymph organs are not complicated by these also being major sites of virus infection. In terms of CD8^+^ T cell immunity, presence of any virus in lymphoid organs was a useful a guide for defining priming sites, even if the amounts measured were poorly predictive of antigen presentation levels. For example, despite very low levels of infectious virus being found in the various LN after i.v. infection, priming was robust at all these sites. Perhaps in this situation many APCs are reached by the ample (1×10^6^ PFU) inoculum and despite undergoing abortive infections, these cells persist long enough to prime effectively. Alternatively there may be a reservoir of antigen that is cross presented, but the evidence thus far suggests that direct priming is more important for VACV-specific CD8^+^ T cells [60]. The kinetics of antigen presentation as determined for the i.d. and i.v. routes were remarkably similar with sustained activation of OT-I cells seen until day 5–6, though it was decreasing by this time. This result rules out the premature loss of antigen presentation in LN, perhaps as a result of killing of APCs by IDE-specific T cells, as a mechanism for increased immunodominance associated with the i.d. route. Further the one organ where presentation seemed to decay the fastest was the spleen after i.v. infection and antigen presentation levels there became very similar for i.d. and i.v. routes by day 3–4. This points to early antigen presentation events being more important in setting the immunodominance hierarchy, possibly reflecting the requirement for only a brief encounter with antigen to drive CD8^+^ T cell responses [66], [67].

We considered the possibility that there was something qualitatively different between the APCs in the spleen and LN that leads to reduced immunodominance. If this were correct, reduced immunodominance should be the hallmark of any responses primed in the spleen. However, reducing virus dose by i.v. and i.p. routes and thereby restricted priming to the spleen and a LN respectively, sharpened immunodominance in both cases. On the other hand, the lower doses also greatly limited the number of APCs available at any priming site, as demonstrated by poor priming of OT-I cells. Strikingly, restricting priming to the mediastinal LN with low dose i.p. infection lead to exactly the same ratio of IDE∶SDE as found for any dose of virus injected i.d. where priming also occurs in a single LN. Further, increasing the number of LNs involved in priming and thereby total APC numbers by infecting multiple peripheral sites did not reduce immunodominance. To reduce immunodominance, a large number of APCs in a single lymph organ were required and this was only provided by the spleen when there was an abundance of antigen. Finally, reducing the virus dose given i.d. did not further sharpen immunodominance, which together with other results here suggests there is a limit to immunodomination. We speculate that the architecture and size of LNs limit APCs and/or some associated essential resources required for priming over a wide range of antigen doses and increase competition between T cells. The possibility of a more competitive environment in LN was confirmed by subjecting VACV-specific CD8^+^ T cells to rivalry from transferred OT-I T cells. The finding that VACV responses were more easily suppressed by OT-I after i.d. but not i.v. infection is consistent with greater competition across specificities when priming is restricted to LN. This leads to the conclusion that immunodomination in LNs, which are the main priming sites after i.d. infection, suppresses responses to SDE.

Having established i.d. infection with VACV as a setting where immunodomination can occur in primary responses, we decided to take advantage of this model to examine the role of costimulation in immunodominance. Our data suggest that increasing levels of costimulators to APCs that directly prime CD8^+^ T cells in a LN reduces the level of immunodomination by the IDE. Conversely, there was no advantage for SDE (or the IDE) when these constimulators were expressed by a virus given by the i.p. route. This effect for the i.d. route might be achieved either by reducing competition for costimulation on individual APCs or possibly extending the number of APCs that have adequate levels of costimulators to prime CD8^+^ T cells. Several groups have demonstrated that CD8^+^ T cell responses can occur in the absence of costimulation via CD80/CD86 and CD28, but that these responses are substantially compromised [58], [68]–[71]. The only report to examine multiple epitopes came to the conclusion that costimulation affected IDE and SDE equally [58]. This is not necessarily inconsistent with our findings or conclusions. We are not suggesting that IDE and SDE have a differential requirement for costimulation, but rather that in the context of priming in LNs, costimulation is limiting and IDE have an advantage in competing for this resource. The VACV strains that express CD80 and CD86 used here were originally developed to be improved vaccine vectors [72]. Other reports with similar viruses suggest that they enhance magnitude and avidity of CD8^+^ T cell responses to model antigens that are co-expressed from the same virus [73], [74]. While we have not tested avidity, our data on magnitude are consistent with these published reports: the epitopes examined previously would rank as SDE and the immunizations published were done using a peripheral route (s.c.). However, the assumption made until now was that all CD8^+^ T cell responses could be boosted by expression of the costimulators. Here we show that the benefit of expressing costimulators, at least in terms of magnitude of response may be for SDE only. This reinforces again the importance of examining responses to multiple epitopes before drawing conclusions about the benefit of different immunization strategies based on pre-clinical models in mice.

In conclusion, we show here that route-related changes in immunodominance after primary infection with VACV are the result of differential spread of virus antigen, which determines the sites of CD8^+^ T cell priming. CD8^+^ T cell priming is more competitive when it is mainly limited to LNs and consequently subdominant specificities are subject to greater immunodomination at these sites. Further, we identify costimulatory molecules as one of the resources that might be limited in LN and therefore drive immunodomination. These data have implications for the interpretation of preclinical vaccinology of vectored vaccines. Beyond these insights this work has ramifications for viral immunology in general, demonstrating clearly the importance of putting responses to any single epitope into the broader context of responses to the whole virus.

## Materials and Methods

### Viruses and cell lines

The majority of viruses used here were kind gifts: WR (Bernard Moss, NIH); VACV NP-S-GFP [54], [75], WR B7-1&B7-2 (called VACV-CD80&86 here) [72] and VSC-8 (as a TK^−^ control virus for VACV-CD80&86) (Jon Yewdell and Jack Bennink, NIH); VACV-ESminigB [48] (S. Tevethia, Penn State Medical College). VACV-fullgB was made by standard homologous recombination methods using plasmid pSC11 [76] to insert the full coding sequence of HSVgB under the control of the p7.5 promoter into the thymidine kinase gene of VACV WR. All recombinant antigens in viruses used here were expressed by the p7.5 promoter from a disrupted thymidine kinase gene of strain WR. Immortalized cell lines, BHK-21 and BS-C-1, were maintained in Dulbecco's Modified Eagle medium (DMEM, Invitrogen) with glutamine and 10% or 2% fetal bovine serum (FBS). VACVs were grown in BHK-21 and purified by centrifugation through a 36% sucrose cushion, then infectivity was titrated on BS-C-1 cells using standard methods.

### Ethics statement

All experiments were done according to Australian NHMRC guidelines contained within the Australian Code of Practice for the Care and Use of Animals for Scientific Purposes and under approvals F-BMB-38.8 and A2011-01 from the Australian National University Animal Ethics and Experimentation Committee.

### Mice and infections

Specific pathogen-free C57BL/6, B6.SJL and OT-I transgenic mice were obtained from the Animal Resource Centre (Perth, Australia) and APF (Canberra, Australia). Unless stated, mice were infected with 1×10^6^ PFU of VACV by i.p., i.v. or s.c. in 200 µl of PBS or by i.d. [77] in 5 or 10 µl of PBS.

### Stimulation with peptides and intracellular cytokine staining

Mice were euthanized 7 days after infection and spleens were taken for analysis of CD8^+^ T cell responses by intracellular cytokine staining (ICS) as described previously [47]. Briefly, splenocytes were plated at 1×10^6^ cells/well into round-bottom 96-well plates. Synthetic peptides (Table 1) were added to a final concentration of 10^−7^ M and plates were incubated at 37°C with 5% CO_2_. After 1 hour, 50 µg/ml brefeldin A (Sigma-Aldrich, St. Louis, MO) was added. Plates were incubated for another 3 hours, then spun at 4°C to remove the medium. Cells were resuspended in 40 µl of 1/150-diluted anti-CD8-PE antibody (clone 53-6.7; BD Biosciences, San Jose, CA). After 30 min incubation on ice, cells were washed, resuspended in 50 µl of 1% paraformaldehyde, and incubated at room temperature for 20 min. After two washes, cells were stained with 40 µl of 1/200-diluted anti-IFN-γ allophycocyanin antibody (clone XMG1.2; BD Biosciences) with 0.25% saponin (Sigma-Aldrich) overnight at 4°C. Cells were washed three times before acquisition using a FACS LSRII (BD Biosciences). Analysis was done in Flowjo software (Tree Star, Ashland, OR). Events were gated for live lymphocytes on forward scatter (FSC) × side scatter (SSC), followed by CD8^+^ cells × IFN-γ. Backgrounds as determined for samples without peptide were usually in the order of 0.1–0.2% and were subtracted from the values presented for test samples.

### Virus titration by plaque assay

Organs harvested from infected mice were homogenized in 1 ml glass homogenizers (Wheaton) then rapidly frozen and thawed 3 times in liquid nitrogen and a 37°C water bath. The homogenized organs were then 10-fold serial diluted in DMEM with 2% FBS before adding to the 6-well plates with BS-C-1 cell monolayer. After 90 min of incubation at 37°C with 5% CO_2_, the virus inoculates were removed and replaced by 2 ml/well of 0.4% Sodium carboxymethyl cellulose (CMC, Sigma-Aldrich) in DMEM with 2% FBS. Plates were incubated at 37°C with 5% CO_2_ for 3 days, then crystal violet (Sigma-Aldrich) used to stain. Plaques were counted and virus titers were determined according to the dilution factor.

### *In vivo* T cell activation and CD69 staining

CD8^+^ T cells were prepared from the spleens and lymph nodes of naive OT-I mice using magnetic bead-based negative selection (Miltenyi), and resuspended in PBS with 2% FBS for i.v. transfer. Each B6.SJL mouse received about 5×10^6^ OT-I CD8+ T cells and rested for >18 hours. 24 hours after infection with 10^6^ PFU of VACV NP-S-GFP by various routes, these infected and naive mice were sacrificed. Single cell suspensions were made from the spleens and lymph nodes, and stained with anti-CD8-PE antibody (clone 53-6.7; BD Biosciences, San Jose, CA), anti-CD45.2-APC antibody (clone 104; BioLegend, San Diego, CA) and anti-CD69-PE-Cy7 (clone H1.2F3; BD Biosciences, San Jose, CA) before acquiring by FACS LSRII (BD Biosciences). The activation of OVA-specific T cells is determined by CD69 expression on CD45.2^+^CD8^+^ cells.

### *In vivo* cytotoxicity assay

Target cells for cytotoxicity detection were prepared from naive C57BL/6 mice and split into two populations for peptide-pulsing with B8_20_ and SIINFEKL respectively at 37°C for 1 hour. These two populations were then labeled with different concentration of CFSE (Sigma-Aldrich), 9 µM for B8_20_ (CFSE^Hi^) and 0.9 µM for SIINFEKL (CFSE^Lo^), before mixing equal number of cells together for i.v. transfer. A total of 2×10^7^ cells were injected into each mouse which was infected with 1×10^6^ PFU of VACV WR for days as described. After 4 hours, the mice were sacrificed for their spleens and lymph nodes. The cell numbers in two CFSE-positive populations were acquired by FACS LSRII (BD Biosciences) for analysis. The specific lysis was calculated by the following formula: [1−(CFSE^Lo^/CFSE^Hi^)_naive_/(CFSE^Lo^/CFSE^Hi^)_infected_]×100.

### Statistical analysis

Unless stated otherwise, statistical comparisons were done using an unpaired t test with Welch's correction because populations had unequal variance. All tests were analyzed with the aid of GraphPad Prism software (GraphPad, La Jolla, CA).

## Supporting Information

Figure S1 **Time course of cytotoxicity in LN and spleen after i.d. infection.** C57BL/6 mice were i.d. injected with 1×10^6^ PFU of VACV WR, and *in vivo* cytotoxicity in lymphoid organs were determined on designated days post-infection by measuring the specific lysis of B8_20_ peptide-pulsed splenocytes. Graph shows the average ± SEM; data are from more than two experiments and three or more mice for each time point. Statistical significance was determined by unpaired Student's t-test.
(TIF)

Figure S2 **Similar immunodominance was observed after infection with VACV NP-S-GFP via various routes.** CD8^+^ T cell responses to 15 VACV epitopes in C57BL/6 mice infected with 1×10^6^ PFU of VACV NP-S-GFP were determined by intracellular staining of IFN-γ. Graph shows the fraction of all measured responses (sum of responses to all 15 VACV peptides) accounted for by B8_20_-specific (IDE) and the sum of SDE-specific CD8^+^ T cells. Data are from two experiments and total mice for each route were: i.v. 6, i.p. 9 and i.d. 6.
(TIF)

Figure S3 **Immunodominance was maintained through the course of infection.** CD8^+^ T cell responses to VACV epitopes B8_20_, A8_189_, K3_6_ and B2_54_ in C57BL/6 mice injected i.p. with 1×10^3^ PFU of VACV WR were determined as previously described on designated day after infection. Graph shows the mean and SEM of the fraction of the total measured response accounted for by B8_20_–specific CD8^+^ T cells. Data are from 3 mice per group on each day, except for day 6 (marked with #) where only 1 of 3 mice had detectable responses to any peptide.
(TIF)

Figure S4 **No significant suppression of VACV-specific responses by OT-I in the absence of OVA_257_ expression.** After being transferred with designated numbers of OT-I CD8^+^ T cells and rested for overnight, B6.SJL mice were infected with 1×10^6^ PFU of control virus VSC-8, which does not express OVA_257_. Seven days later, CD8^+^ T cell responses to 15 VACV epitopes plus OVA_257_ were measured by intracellular staining of IFN-γ. Graph shows the average ± SEM of sum of VACV-specific responses in percent of CD8^+^ cells (left) or total number of CD8^+^ cells per spleen (right).
(TIF)
