# Supplementary figures and images for: Immunodomination during Peripheral Vaccinia Virus Infection
Source: PLoS Pathog. 2013 Apr 25;9(4):e1003329. doi: 10.1371/journal.ppat.1003329 (PMC3635974; doi:10.1371/journal.ppat.1003329)

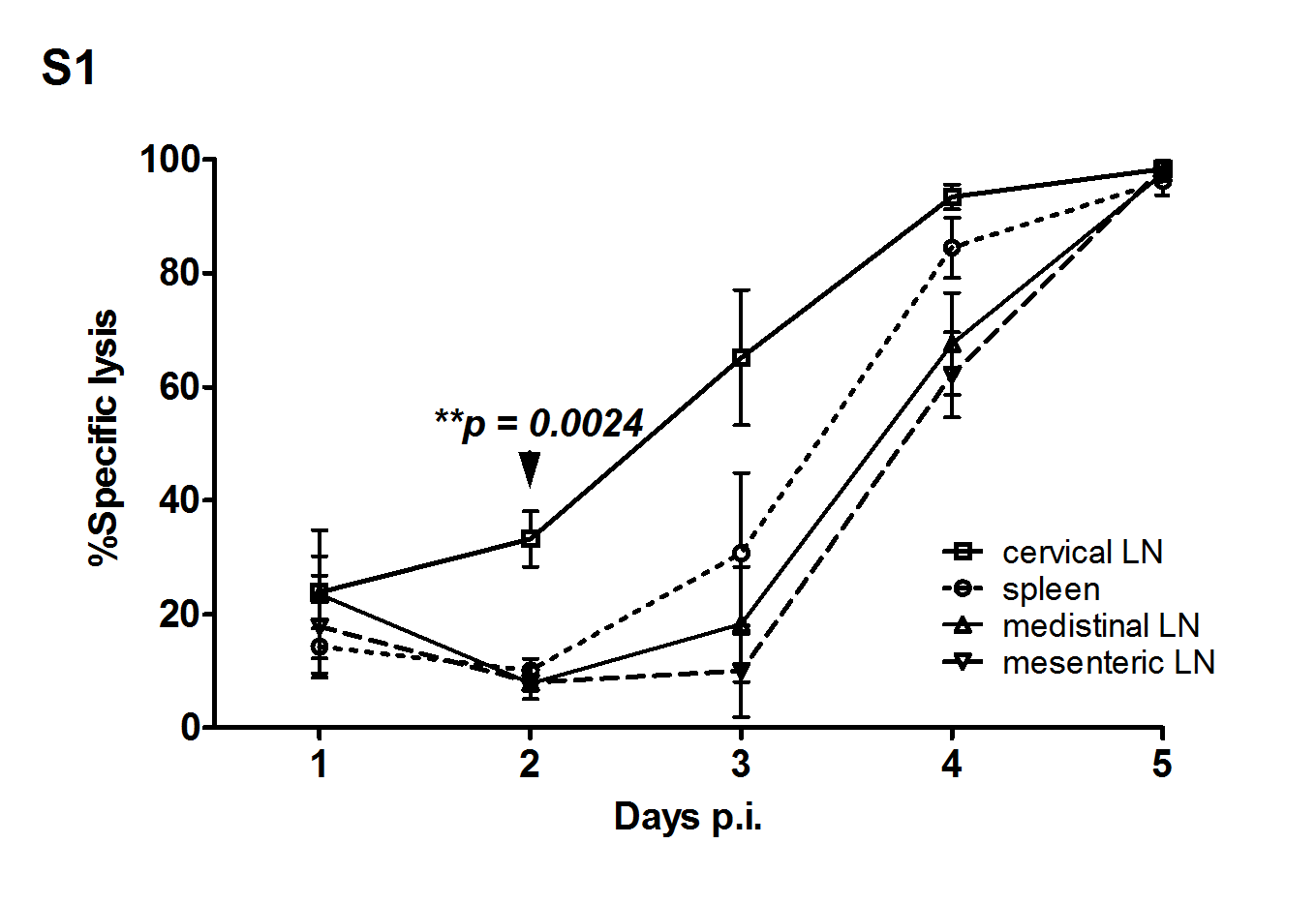

Supplement: Figure S1 — Time course of cytotoxicity in LN and spleen after i.d. infection. C57BL/6 mice were i.d. injected with 1×10^6^ PFU of VACV WR, and in vivo cytotoxicity in lymphoid organs were determined on designated days post-infection by measuring the specific lysis of B820 peptide-pulsed splenocytes. Graph shows the average ± SEM; data are from more than two experiments and three or more mice for each time point. Statistical significance was determined by unpaired Student's t-test. (TIF) [file ppat.1003329.s001.tif]

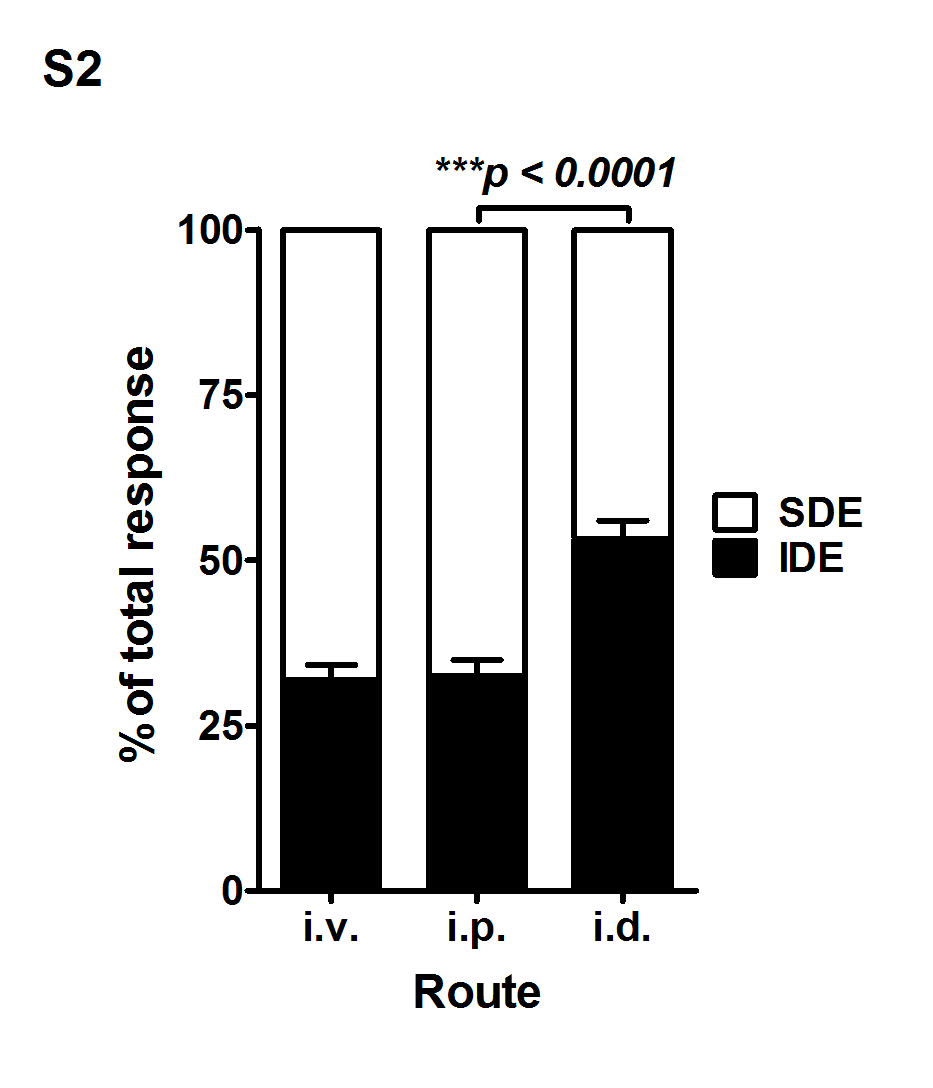

Supplement: Figure S2 — Similar immunodominance was observed after infection with VACV NP-S-GFP via various routes. CD8+ T cell responses to 15 VACV epitopes in C57BL/6 mice infected with 1×10^6^ PFU of VACV NP-S-GFP were determined by intracellular staining of IFN-γ. Graph shows the fraction of all measured responses (sum of responses to all 15 VACV peptides) accounted for by B820-specific (IDE) and the sum of SDE-specific CD8+ T cells. Data are from two experiments and total mice for each route were: i.v. 6, i.p. 9 and i.d. 6. (TIF) [file ppat.1003329.s002.tif]

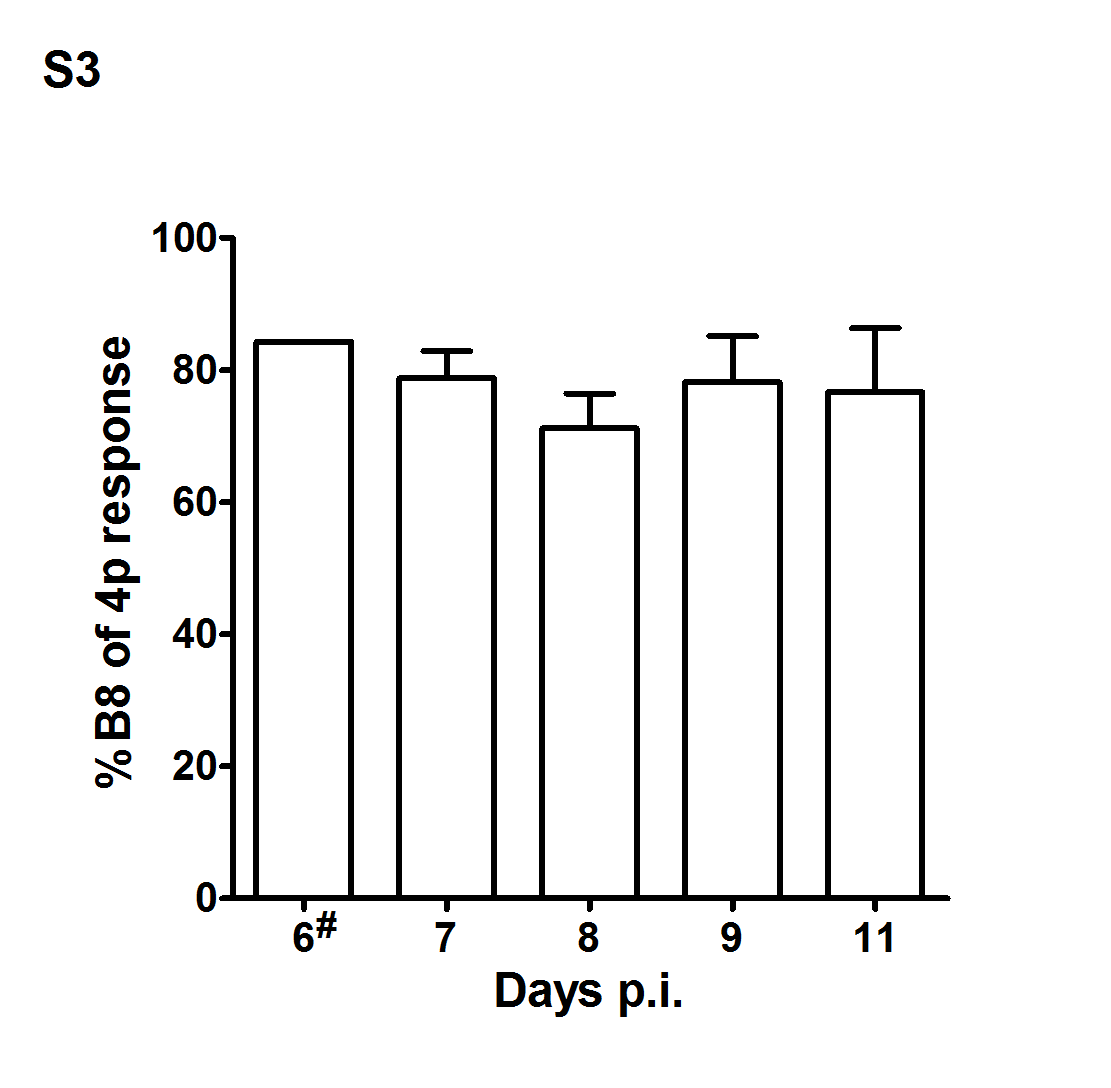

Supplement: Figure S3 — Immunodominance was maintained through the course of infection. CD8+ T cell responses to VACV epitopes B820, A8189, K36 and B254 in C57BL/6 mice injected i.p. with 1×10^3^ PFU of VACV WR were determined as previously described on designated day after infection. Graph shows the mean and SEM of the fraction of the total measured response accounted for by B820–specific CD8+ T cells. Data are from 3 mice per group on each day, except for day 6 (marked with #) where only 1 of 3 mice had detectable responses to any peptide. (TIF) [file ppat.1003329.s003.tif]

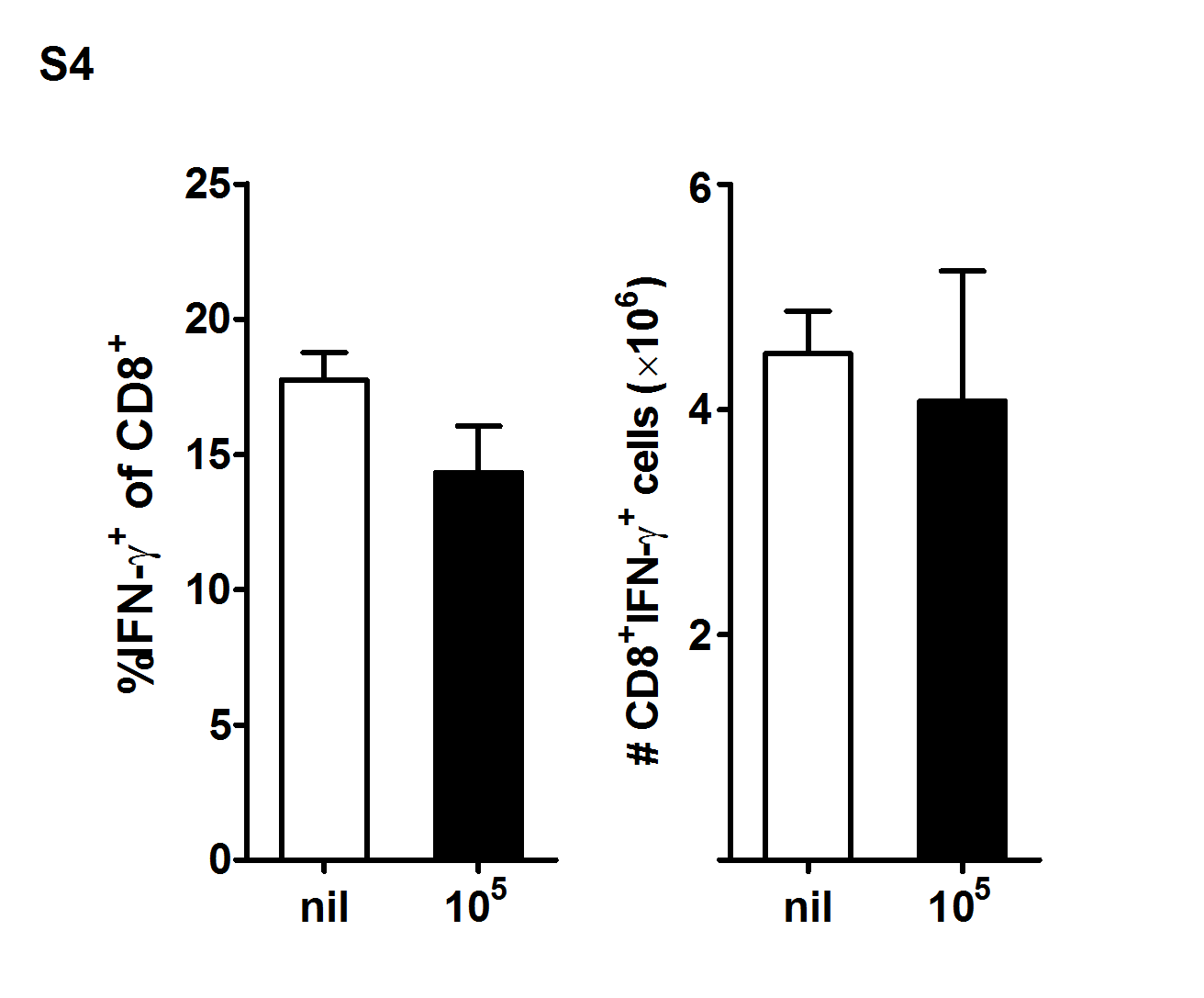

Supplement: Figure S4 — No significant suppression of VACV-specific responses by OT-I in the absence of OVA257 expression. After being transferred with designated numbers of OT-I CD8+ T cells and rested for overnight, B6.SJL mice were infected with 1×10^6^ PFU of control virus VSC-8, which does not express OVA257. Seven days later, CD8+ T cell responses to 15 VACV epitopes plus OVA257 were measured by intracellular staining of IFN-γ. Graph shows the average ± SEM of sum of VACV-specific responses in percent of CD8+ cells (left) or total number of CD8+ cells per spleen (right). (TIF) [file ppat.1003329.s004.tif]
